# Mixed methods evaluation of targeted case finding for cardiovascular disease prevention using a stepped wedged cluster RCT

**DOI:** 10.1186/1471-2458-12-908

**Published:** 2012-10-26

**Authors:** Tom Marshall, Michael Caley, Karla Hemming, Paramjit Gill, Nicola Gale, Kate Jolly

**Affiliations:** 1 School of Health and Population Sciences, University of Birmingham, Edgbaston, Birmingham, B15 2TT, UK; 2 Warwickshire Primary Care Trust, Westgate House, Market Street, Warwick, CV34 4DE, UK

**Keywords:** Statinsm, Cardiovascular diseases, Prevention, Risk factors, Cluster randomised controlled trial

## Abstract

**Background:**

A pilot project cardiovascular prevention was implemented in Sandwell (West Midlands, UK). This used electronic primary care records to identify untreated patients at high risk of cardiovascular disease then invited these high risk patients for assessment by a nurse in their own general practice. Those found to be eligible for treatment were offered treatment. During the pilot a higher proportion of high risk patients were started on treatment in the intervention practices than in control practices. Following the apparent success of the prevention project, it was intended to extend the service to all practices across the Sandwell area. However the pilot project was not a robust evaluation. There was a need for an efficient evaluation that would not disrupt the planned rollout of the project.

**Methods/design:**

Project nurses will sequentially implement targeted cardiovascular case finding in a phased way across all general practices, with the sequence of general practices determined randomly. This is a stepped wedge randomised controlled trial design. The target population is patients aged 35 to 74, without diabetes or cardiovascular disease whose ten-year cardiovascular risk, (determined from data in their electronic records) is ≥20%. The primary outcome is the number of high risk patients started on treatment, because these data could be efficiently obtained from electronic primary care records. From this we can determine the effects of the case finding programme on the proportion of high risk patients started on treatment in practices before and after implementation of targeted case finding. Cost-effectiveness will be modelled from the predicted effects of treatments on cardiovascular events and associated health service costs. Alongside the implementation it is intended to interview clinical staff and patients who participated in the programme in order to determine acceptability to patients and clinicians. Practical considerations meant that 26 practices in Sandwell could be randomised, including about 6,250 patients at high risk of cardiovascular disease. This gives sufficient power for evaluation.

**Discussion:**

It is possible to design a stepped wedge randomised controlled trial using routine data to determine the primary outcome to evaluate implementation of a cardiovascular prevention programme.

## Background

Cardiovascular disease (CVD) remains the UK’s largest cause of morbidity and mortality. There is overwhelming evidence that drug treatment to lower blood pressure and lipid levels reduce the incidence of and mortality from CVD. Under UK guidelines, statins are recommended for all high-risk patients and antihypertensive drugs for those high risk patients if their blood pressure is ≥140/90 mm Hg [1-4]. They also recommend that patients with familial hypercholesterolaemia are prescribed statins and those whose blood pressure ≥160/100 mm Hg are prescribed antihypertensives, irrespective of their risk status [1,3,5]. The guidelines define high risk as either the presence of atherosclerotic disease or ≥20% predicted ten-year cumulative risk of coronary heart disease or stroke, using the Framingham equations. [5] This means that all those at ≥20% ten-year CVD risk are eligible for statin treatment and many are eligible for both statin and antihypertensive treatments. Some guidelines also recommend that individuals at ≥20% ten-year CVD risk should be offered aspirin [3,4]. However, the recommendation is controversial as the risk benefit ratio in patients taking other preventive treatments is only marginally favourable [6].

Historically in the UK there have not been systematic attempts to identify and offer CVD prevention and most primary prevention of CVD has been undertaken when patients are identified opportunistically in primary care. However there may be weaknesses with this approach. GPs do not always follow guidelines in routine clinical practice [7]. They may not fully recognise the clinical importance of statin prescribing and when eligible patients consult, physicians commonly delay prescribing decisions until the next visit [8,9]. There is also confusion about which patients are eligible for treatment. In New Zealand, where risk based hypertension guidelines were first adopted, clinicians reported they would treat low risk younger adults at lower blood pressures than high risk older adults [10]. When deciding whether to prescribe, GPs also seem to give greater weight to individual risk factor values than global risk [11]. In opportunistic screening CVD risk calculation may also not be carried out, it may be done incorrectly and it often varies from one physician to another [12-15]. The opportunistic strategy therefore may not be sufficient and in the UK, many eligible individuals remain untreated. In 2009, 29% of adults were hypertensive of whom 51% were untreated and the figures have changed little since 2005 [16].

Recently, the NHS announced a programme of NHS Health Checks, to be offered to all adults aged 40 to 74 as part of a five year rolling programme to screen people for CVD and diabetes and offer lifestyle and preventative advice [17]. Economic modelling suggested that universal screening, such as that implemented under the NHS health checks, would be cost effective [18]. However, the modelling did not explicitly compare a strategy of universal screening of all those in the selected age range with case finding targeted at those most likely to benefit. Furthermore, cost-effectiveness modelling studies indicate that a strategy of prioritising untreated patients for assessment on the basis of their estimated ten-year CVD risk should, in theory, be the most efficient prevention strategy for untreated patients [19,20]. This finding has further support from a subsequent modelling study using a different multivariable risk prediction equation [21].

Economic modeling studies therefore suggest that targeted, rather than universal, screening approaches could be cost-effective. However, since it was uncertain whether results of modeling studies would be realised in actual practice, we are therefore undertaking a controlled evaluation of a targeted versus opertunistic screening approach for cardiovascular risk.

This paper presents the protocol for a stepped wedged cluster randomised controlled trial that aims to quantify the benefits of targeted case finding of patients at high risk of CVD over opportunistic assessment and overcome the challenges of evaluation. The primary hypothesis is that targeted case finding will increase the rate at which high risk patients are started on treatment. A pilot study, reported in full elsewhere, was undertaken to determine feasibility and for completeness the findings from this pilot study are briefly summarised before the methodology for the full study are presented [22].

### Pilot study for targeted case finding

In 2005–6 a pilot project of case finding for untreated high risk patients was implemented in six general practices in the area covered by Sandwell Primary Care Trust in the West Midlands, England. In six general practices cardiovascular risk factor data were extracted from electronic primary care records of all untreated patients aged 35 to 74 and high risk patients were identified from information in their records. Over the course of one year, in four of the six practices, high risk patients were sent invitation letters offering assessment by the project nurse in their own general practice by a project nurse and if, they were confirmed to be eligible for treatment were referred to their GP for treatment. Over the same period, two control general practices were provided with an equivalent list identifying their high risk patients but elected to assess patients opportunistically.

Twice as many high-risk patients had their CVD risk factors assessed in the intervention than control practices (62% v 28%). Four fifths of those patients who were assessed, were confirmed to be high risk. Three times more eligible high-risk patients were started on preventive treatment in the intervention than in the control practices (41% v 13%) [22].

Results from the Sandwell pilot suggested that it is feasible to implement a programme of targeted case finding and that this might have similar advantages over opportunistic screening to those estimated from modelling studies [19,20]. A further study, in Solihull Care Trust, also demonstrated support for feasibility of implementation of the intervention using a prescribing advisor instead of a nurse.

However, it was not known if these results could be replicated across a larger number of practices and the small and non-randomised nature of the evaluation means that the apparent increase in patients started on treatment could reflect systematic differences between the practices rather than effects of the intervention. There was also little information on the experience of the case finding programme from the perspective of patients or of practice staff.

### Challenges of implementation and evaluation

The evidence of effectiveness of the intervention from the pilot study, although encouraging, is limited and so it is important to undertake a robust evaluation of the programme. If targeted case finding could be demonstrated to be more effective than opportunistic assessment it could be advocated more widely across primary care systems. However, both implementation and evaluation presented a number of challenges. First, an efficient method must be found to identify untreated high risk patients for invitation. Second, a practical plan was needed for implementation across a large number of general practices given limited resources for implementation. Third, a methodology was needed to evaluate the effects of targeted case finding given the fact that in both Sandwell and Solihull the case-finding programmes were to be implemented in all practices at the same time. Fourth, an efficient means of data collection needed to be identified.

### Implementation and stepped wedge evaluation

Within Sandwell Primary Care Trust there was support to continue and expand the targeted case finding project to include all its practices and within Solihull Care Trust there was support to expand the project to include all practices in the northern part of the care trust. However the number of project nurses/prescribing advisors available to implement the case finding was limited and it was therefore not possible to offer targeted case finding to all practices concurrently. It was therefore planned that the project nurses/prescribing advisors would sequentially implement the target case finding in a phased way across all general practices. Because the unit of intervention for the case finding programme was a general practice, it was neither practical nor acceptable to randomly allocate individual patients to invitation to a case finding programme or usual care. However the phased intervention across general practices provided an opportunity to evaluate the effects of the case finding programme on the proportion of high risk patients started on treatment in practices before and after implementation of targeted case finding. If the order in which case finding was implemented in practices could be randomised this would form the basis of a stepped wedged cluster randomised controlled trial [23].

**Figure 1:**
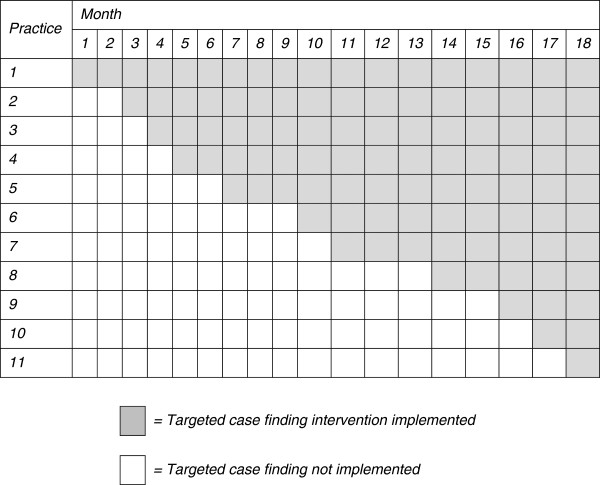
Stepped wedge trial design with different steps of different durations.

A stepped wedge cluster randomised controlled trial is a study design that has been used to evaluate interventions in health care, social policy and education [24]. They have mainly been used to evaluate routine implementation and the earliest published example involved roll out of Hepatitis B immunisation across regions of Gambia [25]. In this type of trial the intervention is implemented in a number of clusters but the order in which clusters receive the intervention is random (Figure 1). At the end of the trial all clusters will have received the intervention. Outcome data are collected at each step; in other words at each time point where a new cluster receives the intervention. Analysis therefore compares data points in the control section of the wedge (not exposed to the intervention) with those in the intervention section (exposed to the intervention).

## Methods/design

### Practice recruitment

By 2008 of 65 practices in Sandwell Primary Care Trust, case finding had already been completed in 12 (six as part of the pilot project); in a further 25, case finding had started or a start date had already been agreed; two practices did not have available consultation rooms in the practice building, leaving 26 practices for randomisation. The total population registered with these practices was approximately 125,000 and from the pilot study it was anticipated that 5% (about 6,250) would be untreated high risk patients.

There were six practices in Solihull Care Trust in whom the targeted case finding programme could be implemented. The total population registered in these practices was 18,938 and it was anticipated that 945 (5%) would be untreated high risk patients.

Therefore overall 32 practices, representing 32 separate clusters, were included in the study with a combined estimated 7,247 untreated people at high risk of CVD.

### Randomisation

Randomisation was stratified by primary care trust. In Sandwell PCT, after discussion with the case finding programme manager, it was agreed that 11 practices were a higher priority for the case finding intervention and the remaining 15 practices were a lower priority. It was therefore decided to stratify the practices into two groups for randomisation: early intervention and late intervention. A table of random numbers was generated to determine the order in which practices should receive the project nurse led intervention in the early intervention group and a second table for practices in the late intervention group.

The six practices in Solihull were of different characteristics, two were single handed and two were located very near each other and served similar populations. They were therefore stratified into three pairs (two single handed, two similar location and two remaining practices) for randomisation. A table of random numbers was generated to determine which of each pair should have the intervention first and which second.

### Inclusion criteria

All patients aged 35 to 74 who were not currently on the CVD or diabetic registers and who were not currently receiving antihypertensive or statin treatment (no prescription within 90 days) were included in the programme. From this were selected all those whose estimated ten-year CVD risk ≥20%.

Practice software systems were used to identify patients with a ten-year CVD risk ≥20%. In Sandwell PCT all practices subscribe to Clinical Manager software manufactured by MSDi [26]. This software produces lists of patients meeting pre-specified criteria (e.g. diabetic patients due for a blood pressure measurement). The manufacturers incorporated an additional module into the software to calculate ten-year CVD risk for all untreated patients without CVD using the Framingham risk equations [27]. The following method was used to deal with missing risk factor data. If smoking status was missing the patient was assumed to be a non smoker; if blood pressure or cholesterol values were missing a default value was used, based on the average for an untreated person of that age, sex, smoking status and diabetic status. This followed a previously described method [28]. In Sandwell PCT this allowed every practice to produce a list of untreated patients at ≥20% ten-year CVD risk (high risk patients).

In Solihull PCT practice data was extracted using the search facility in the electronic patient records and exported to an Excel spreadsheet. Ten year CVD risk (high risk patients) was calculated in the spreadsheet using the same method and patients at ≥20% ten-year CVD risk (high risk patients) were identified for the practices.

### Exclusion criteria

Participating practices reviewed the list of high risk patients identified and excluded those who had died, moved practice and or who the GP judged were unsuitable for CVD prevention (e.g. those with a terminal illness).

### Intervention

The intervention in Sandwell was delivered by project nurses trained in the management of either diabetes or coronary heart disease. In Solihull prescribing advisors, trained pharmacists who were licensed to prescribe, delivered the intervention.

In the intervention practices the project nurse (Sandwell) or prescribing advisor (Solihull) arranged for a letter along with an information sheet about the case finding project to be sent from the GP inviting patients for cardiovascular assessment. The letter specifically mentioned that patients might be offered lifestyle advice and medication ( Additional file 1 and Additional file 2). The patient was given the time and date of an appointment for their CVD assessment and asked to reschedule if this was unsuitable. If they did not attend or reschedule the appointment two further attempts were made to contact patients either by letter or telephone.

Patients who attended underwent a full cardiovascular risk factor assessment, including enquiry about smoking, measurement of blood pressure, blood tests for fasting glucose, total cholesterol and HDL cholesterol. Each patient’s ten-year CVD risk was calculated using the Framingham equation as recommended in UK guidelines based on their measured risk factors and their eligibility for treatment was determined [2,3]. If they were eligible for antihypertensive or statin treatment the project nurse/prescribing advisor discussed the risks and benefits of treatment with the patient and the general practitioner was informed so that treatment could be initiated. If appropriate the patient could also be referred to local smoking cessation services, for advice on physical activity or for dietary advice about weight loss.

Patients at highest risk were invited first followed by those at next highest risk until all high-risk patients (≥20% ten-year CVD risk) had been invited. The project nurse (or prescribing advisor) then repeated the process in the next practice in a sequence determined by the randomisation. Because the practices varied in size this meant that the number of weeks spent at each practice would be expected to vary from one practice to another. It was anticipated that it would take about 18 months to complete the implementation of targeted case-finding in all Sandwell practices and about 12 months in Solihull practices.

### Primary outcomes

Since there is good evidence for the effectiveness of CVD prevention, it is reasonable to use uptake of treatment as evidence of effectiveness. Prescriptions of antihypertensive drugs or statins are recorded in electronic primary care records, therefore routine data could be used to assess outcomes.

The primary outcome is the number of high-risk patients started on at least one preventive treatment: an antihypertensive drug or a statin. Antihypertensive drugs and statins are defined by the chapter codes in the British National Formulary [29].

Secondary outcomes include the number of patients who have cardiovascular risk factors assessed (blood pressure, lipid levels); the number of patients referred to services for lifestyle advice (smoking cessation services, advice on physical activity, dietetic advice on weigh loss); changes cardiovascular risk factors (blood pressure, lipid levels); cardiovascular events (new diagnoses of angina, MI, TIA or stroke).

### Follow up and data collection

Data extraction will be undertaken as a single data extract at least one year after targeted case-finding has been implemented in the last practice. This allows for at least a year of follow up. The number of untreated high-risk patients who are started on treatment will be assessed at the start of each step in the stepped wedge trial. An outline of the data extracted is given in Table 1.

**Table 1:** Data to be extracted from electronic primary care records at the end of the study

**Data to be extracted**	
Patient characteristics	Age
	Sex
	Ethnicity
	Date of death,
	Date of leaving practice
Diagnoses and dates of diagnoses	Diabetic status
	Smoking status
	Angina
	MI
	TIA
	Stroke
	Peripheral vascular disease
	Death
Cardiovascular risk factor status and dates	Smoking status
	Systolic blood pressures
	Diastolic blood pressures
	Total cholesterol
	HDL cholesterol
	Family history of premature coronary heart disease
	Left ventricular hypertrophy
Prescriptions and dates of prescriptions	BNF Chapter 2.2.1 Thiazide and related diuretics
	BNF Chapter 2.2.3 Potassium-sparing diuretics and other diuretics
	BNF Chapter 2.2.4 Potassium-sparing diuretics with other diuretics
	BNF Chapter 2.4 Beta-blockers
	BNF Chapter 2.5.1 Vasodilator antihypertensives
	BNF Chapter 2.5.3 Adrenergic neurone blocking drugs
	BNF Chapter 2.5.4 Alpha blockers
	BNF Chapter 2.5.2 Centrally acting antihypertensives
	BNF Chapter 2.5.5.1 ACE inhibitors
	BNF Chapter 2.5.5.2 ARB-2
	BNF Chapter 2.6.2 Calcium-channel blockers
	BNF Chapter 2.8.1 Parenenteral anticoagulant
	BNF Chapter 2.8.2 Oral anticoagulant
	BNF Chapter 2.9 Antiplatelet drug
	BNF Chapter 2.12 Lipid lowering drug
	Over the counter aspirin use

### Analysis

The characteristics of the populations served by the GP practice will be summarised, including, primary care trust, list size, age of practice population, gender, ethnicity and practice Index of Multiple Deprivation score. [30] These characteristics will be summarised by numbers and percentages, means and standard deviations or medians and inter-quartile ranges as appropriate. We will then report the number of patients excluded in the study by each of the inclusion criteria (age, CVD status and medication use), and also the number of patients meeting the study inclusion criteria.

Fidelity of uptake will be explored by reporting, the number of patients invited, the number of patients seen by the nurse/prescribing advisor and the proportion who were subsequently confirmed to have a CVD risk ≥20%.

The characteristics of the patients included in the invited population will also be summarised. These characteristics will include gender, age, blood pressure, lipids, glucose, medication use (at baseline), cardio-vascular disease, smoking status, Framingham risk score, and referrals to smoking cessation, physical advice and dietetic advice services. These characteristics will be summarised by their values at baseline (2008) and will be compared between those not attending; and for those attending stratified by whether their calculated risk was greater than 20%.

The primary aim of the study is to evaluate whether there is a difference in proportion of eligible patients on the relevant prescription medication before and after the practice was exposed to the intervention. In statistical terms this null-hypothesis (no difference) can be tested using a mixed logistic regression model with binary outcome (prescription of the appropriate medication). Important independent variables to consider are the clustering effect (i.e. effect of GP practice), calendar time effect (since the intervention is sequentially rolled-out) and an indicator of intervention exposure for each practice at each time point; in an to adjustment for other characteristics [4]. These models will be fitted using population averaged models using GEE methods in STATA, allowing for clustering and adjusting for individual level covariates and any cluster level covariates where available. Population averaged models as opposed to random effects models (also known as marginal models) will be used as within the framework of cluster randomised trials, random effects models both lack appropriate interpretation and might be biased [31]. The covariates to be included in the adjustment will be pre-specified and will include practice size and patient level characteristics (age, sex, diabetic status, smoking status, blood pressure, total cholesterol and HDL cholesterol); GP practice will be included as a random effect; time point as a fixed effect; and exposure or non-exposure as the main intervention effect. Null hypotheses for secondary outcomes take a similar form to that for the primary outcome. Analysis of the secondary outcomes will take a similar form to that described for the primary outcome.

The primary outcome will be considered significant at the 5% level and so 95% confidence intervals (CIs) reported; whereas secondary outcomes will be deemed significant at the 1% level (and so 99% CIs reported). This difference in levels of significance, give more weight to the primary outcomes. Analysis will be stratified by primary care trust: the analysis of the project nurse led intervention in Sandwell and the prescribing advisor led intervention in Solihull will be carried out separately.

### Sample size & number of practices

Since the analysis is to be stratified by primary care trust sample size calculations were also stratified by trust. However, since this is a pragmatic evaluation the study has a limited sample size to those practices which agreed to participate in the evaluation. In the Solihull trust only 6 practices agreed to participate and it was not expected that this would provide adequate power. We therefore based power calculations on the Sandwell part of the study only. In the pilot study approximately 5% to 6% of registered patients in each practice were found to be untreated high risk patients. In Sandwell this means we expect to invite between 6,241 and 7,489 untreated high-risk patients from a total registered population of 124,820. From this fixed study size, it is possible to estimate the difference that will be detectable (difference between patients exposed and not exposed to the intervention in proportion of eligible patients medicated). These patients are spread across 26 clusters, each with a conservative estimate of average size of 240 (assuming 5% of patients eligible); but with some variation between practices sizes with coefficient of variation of 0.74.

The difference detectable depends on the level of intra-cluster correlation (ICC), the variation in sizes of practices, and the proportion of patients medicated in the control arm. Estimates of ICC would ideally come from other similar studies, but in the absence of such evidence we are guided by a review of estimates of ICCs which found that ICC values are typically between 0.02 and 0.1 [32]. The stepped wedge nature of this study should mean that impact of intra-cluster correlations are lower than in conventional cluster trials [4], so these values can be viewed as conservative. The pilot study estimated that the proportion of eligible patients started on medication over the course of one year is around 13%.

So, using several different estimates of likely values of ICCs and current proportions of patients medicated, we have estimated what values for outcomes post intervention could be detected (all for 80% power, 5% significance and coefficient of variation of 0.74). We have used conventional cluster RCT power calculation methods since this study is a modification of the conventional stepped wedge design and so provide conservative estimates [33].

For example, if the current proportion of patients medicated is about 13%, and values for intra cluster correlation low (ICC of 0.01) then this study would be powered to detect an increase in proportion of high risk patients started on treatment to 19% (a relative risk of 1.46); with an ICC of 0.05 the study would detect an increase to 21% (relative risk 1.62); with an ICC of 0.1 the study would detect an increase to 26% (relative risk 2.00). If however the current proportion of patients medicated was closer to 20% and the ICC much higher (say 0.1) then this study would provide 80% power to detect an increase to 40% (that is almost a 20% absolute percentage increase).

### Economic evaluation

An economic evaluation will also be carried out. Costs will be imputed from prescribing and consultation rates determined from data extracts. A Markov model will be constructed to determine long term impacts on costs and health outcomes (QALYs) using the effects of treatment in the short term to determine the long term impact on QALY life expectancy and health service costs.

### Qualitative research

Alongside the trial, qualitative research will explore the barriers and facilitators to the implementation of the programme. In combination, the qualitative and quantitative components of the full study will generate knowledge not only on the potential value, but also the feasibility and acceptability, of rolling out the case-finding intervention more widely in the NHS. Birmingham and the Black Country provide the opportunity to explore attitudes to and experience of primary prevention within an ethnically and economically diverse urban community. These findings may also be transferable to other similar programmes for prevention of serious disease in primary care. Interview data will be collected from the implementers of the programme, including the research team, and from patients that have attended.

The aims are:

– To explore the implementation process, using in-depth interviews, and propose theories about what implementation configurations need to be in place for successful implementation.

– To explore the patients’ experience of the intervention and how it fits into their broader health beliefs and health behaviours, using subject-produced photo elicitation [34] (Harper 2002) in the context of an open-ended interview. This will enable an appraisal of the acceptability of the intervention along with insights into barriers and facilitators to adherence.

### Ethical approval

Advice on the need for ethical approval was sought from the National Research Ethics Service. Implementation of the targeted case-finding programme was being rolled out as rapidly as was practical. The order in which the implementation was being carried out in each practice had been determined in advance by randomisation. No additional investigations or measurements were being undertaken and no patient identifiable data would leave the NHS. We were therefore advised that this did not need ethical approval as it was evaluation of a service development [ Additional file 3].

Although interviews with patients and clinical staff for the qualitative evaluation could also be considered as part of the service evaluation we sought and obtained ethical approval from the University of Birmingham ethics committee for this part of the study [ Additional file 4]. Information sheets and consent forms were provided for interview participants who were clinical staff or patients [ Additional file 5, Additional file 6, Additional file 7 and Additional file 8].

## Discussion

There is very limited published evidence for the benefit of targeted case finding for CVD over opportunistic case finding. The prevention and early treatment of CVD, as the biggest killer and a major cause of morbidity in the UK, has the potential to make huge impacts to the health of populations. We have presented the design and methods for a stepped wedged cluster RCT to assess the benefits of systematic case finding of patients at high risk of CVD over opportunistic assessments.

There were strong imperatives to implement the cardiovascular prevention project across all practices due to a widespread consensus that this type of case finding approach “made sense” and the benefits of early intervention in CVD and diabetes are well known. In addition the practices served deprived populations where the burden of cardiovascular disease was relatively high compared to the rest of the country and clinicians felt a responsibility to give their patients these opportunities to improve their health. The mandate to implement case finding in all practices meant it was not possible to conduct a cluster randomised controlled trial, however the stepped wedged cluster design allows the process of implementation to be structured in such a way as to ensure that an evaluation of targeted versus opportunistic case finding could be undertaken.

The limitations that are present in this study, were largely as a result of the need to work with the enthusiasm that existed to implement this approach and evaluate a real world roll out of this approach in the most robust way possible. The study was implemented at a time when the NHS were rolling out health checks in those aged 40 to 74. This might induce contamination, whereby those practices included in this study might also have rolled out the NHS health checks. The study also was limited in its size to those primary care practices which agreed to participate.

One further limitation of this study is the relatively small number of clusters included. The MRC guidelines suggest that a minimum of 10 clusters should be used in each arm of a cluster randomized trial [35]. In this setting we are limited by the number of GP practices in the two primary care trusts. However, the stepped wedge nature of this design, in which all practices will eventually receive the intervention, but the order in which they do so will be randomised, does provide a more robust design over the conventional cluster randomized trial and so may mitigate some of the potential issues surrounding the inclusion of what might be considered as a small number of clusters. This is because within a stepped wedge design each clusters acts as its own control (during the period in which the cluster is not receiving the intervention) and it has been shown that magnitudes of intra-cluster correlations are less important [36]. Furthermore, this study is a modification of the common step-wedge design because after completion of case finding the intervention is not maintained in the clusters (rather the nurse who delivers the intervention moves to the next practice). In effect, this probably means a reduced level of power to detect an effect of the intervention due to contamination of patients not exposed but nonetheless contributing to the analysis in later wedges.

Routine data on prescription of antihypertensives and statins is a pragmatic and easily collected outcome. However this leaves the study vulnerable to primary non compliance as not all those who are issued prescriptions take them.

We hope to be able to determine whether case finding is more effective and cost effective in achieving higher rates of uptake of preventative advice and treatment; whether it results in a reduction in outpatient referrals and admissions to hospital for CVD related events; and whether it is acceptable to clinicians and patients.

## Competing interests

The author(s) declare that they have no competing interests.

## Authors’ contributions

TM developed the original idea to implement and evaluate targeted case finding. MC contributed to the pilot study and to design of the evaluation. KH undertook sample size calculation and contributed to the study design and analysis plan. NG contributed to the qualitative aspects of the study design and analysis. KJ and PG contributed to design of the evaluation. All the authors critically reviewed the paper. All authors read and approved the final manuscript.

## Pre-publication history

The pre-publication history for this paper can be accessed here:

http://www.biomedcentral.com/1471-2458/12/908/prepub

## Supplementary Material

Additional file 1 Suggested letter for practices to send to patients identified to be at high risk of CVD. Click here for file

Additional file 2 Patient information sheet. Click here for file

Additional file 3 One page summary of evaluation-enterprise vault archived item. Click here for file

Additional file 4 Application for ethical review ERN_10-0429. Click here for file

Additional file 5 Consent form. Click here for file

Additional file 6 Consent form. Click here for file

Additional file 7 Participant information sheet. Click here for file

Additional file 8 Participant information sheet. Click here for file
