# Supplementary material for: Mixed methods evaluation of targeted case finding for cardiovascular disease prevention using a stepped wedged cluster RCT
Source: BMC Public Health. 2012 Oct 26;12:908. doi: 10.1186/1471-2458-12-908 (PMC3505746; doi:10.1186/1471-2458-12-908)
Supplement: Additional file 1 — Suggested letter for practices to send to patients identified to be at high risk of CVD. [file 1471-2458-12-908-S1.pdf]

Suggested letter for practices to send to patients identified to be at high risk of CVD

Letter to be sent with the information sheet

Dear

Invitation to attend for a check up on [Date and Time]

As part of our practice's commitment to improving the health of our patients, people aged 35-74 who might be at increased risk of developing heart disease or stroke are being invited to have a check-up.

The check-up will involve:

- checking your blood pressure
- taking a small amount of your blood to check your cholesterol level
- measuring your height and weight
- asking about your medical history
- asking if you smoke
- asking about exercise and diet

We would like to invite you to attend the surgery for one of these 'heart health' check-ups.

At the check-up you will see [name of assessor], a [pharmacist / nurse] who specialises in heart disease prevention. He will offer you advice and answer any questions you may have. If any treatment or follow-up appointments are needed, this will be arranged for you.

An appointment has been arranged for you:

Date:

Day:

Time:

Place

If you cannot make this appointment, please contact the surgery on XXX XXXX to arrange another time.

Yours sincerely

Dr XXXXXX
