# Supplementary material for: Mixed methods evaluation of targeted case finding for cardiovascular disease prevention using a stepped wedged cluster RCT
Source: BMC Public Health. 2012 Oct 26;12:908. doi: 10.1186/1471-2458-12-908 (PMC3505746; doi:10.1186/1471-2458-12-908)
Supplement: Additional file 2 — Patient information sheet. [file 1471-2458-12-908-S2.pdf]

Patient information sheet

Questions and Answers

Why have I been asked for a check up?

Experts recommend that people should have their blood pressure and their cholesterol levels checked. This is because people who feel well can have a high blood pressure or a high cholesterol level without knowing. Experts also recommend that people should be given advice on smoking, exercise and diet.

Is everyone being asked for a check up?

No. The people who have been asked to attend are older people or people who have other risk factors such as smoking, high cholesterol or diabetes. These people are more likely to need advice or treatment.

Who will I see?

You will see a cardiovascular risk nurse who is working with your doctor to help identify and treat people who may be at risk from cardiovascular disease. The nurse will also be working with people at Birmingham University to find out if this is the best way of identifying and treating people who may be at risk of cardiovascular disease.

Who will have access to my medical details?

Your doctor and the nurse who will be treating you.

Information used to find if this approach is the best way is anonymous and cannot be traced to you. If you require further information about the project you can contact Dr Tom Marshall at the Unit of Public Health, Epidemiology and Biostatistics, University of Birmingham, B15 2TT. Tel 0121 414 7832 E-mail: T.P.Marshall@bham.ac.uk

What sort of tablets could I be given?

You could be given a tablet to reduce the amount of cholesterol (fat) in the blood. Usually this is simvastatin.

You could be given a tablet or tablets to lower your blood pressure.

You could be given a tablet to stop your blood getting too sticky and likely to develop clots. Usually this is Aspirin.

Will I need to see the doctor as well?

No. Most people will be seen by the nurse and can be followed up by the nurse. Your doctor will be reviewing the results and it will be your doctor that prescribes medicines for you, but in most cases the doctor will not need to see you in person.

Do I need to attend?

You are not obliged to but we would strongly recommend that you do take up this opportunity because by carrying out some simple tests it is possible to identify and treat things that could lead to a heart attack or stroke. Treated early enough it may help you to live a longer and healthier life. If however you decide not to attend, your doctor will continue to look after you in the usual way and your relationship with your doctor will not be affected.

You will also be invited to talk to somebody about your experiences within the service, but you are not obliged to contribute in this way.
