# Supplementary material for: Mixed methods evaluation of targeted case finding for cardiovascular disease prevention using a stepped wedged cluster RCT
Source: BMC Public Health. 2012 Oct 26;12:908. doi: 10.1186/1471-2458-12-908 (PMC3505746; doi:10.1186/1471-2458-12-908)
Supplement: Additional file 3 — One page summary of evaluation-enterprise vault archived item. [file 1471-2458-12-908-S3.pdf]

From NRES Queries Line **Date** 08 June 2009 12:12:48
To Tom Marshall
Cc
Subject RE: One page summary of evaluation

Your query was reviewed by our Queries Line Advisers.

Dear Tom

Thank you.

I see this as a pragmatic design and given that the intention is to provide the intervention in all practices eventually and as rapidly as possible but use the "timetabling " to study and assess the intervention , I'd classify this as a service evaluation.

Hugh

Streamline your research application process with IRAS (Integrated Research Application System). To view IRAS and for further information visit www.myresearchproject.org.uk

Queries Line
National Research Ethics Service
National Patient Safety Agency
4-8 Maple Street
London
W1T 5HD

Website: www.nres.npsa.nhs.uk
Email: queries@nres.npsa.nhs.uk

Ref: 04/02
**

Help save paper - do you need to print this email?
This e-mail (and any files transmitted with it) is intended for the recipient only. It may contain confidential information and may be protected by law as a legally privileged document and copyright work; its content should be not disclosed, forwarded or copied. If you are not the intended recipient, any reading, printing, storage, disclosure, copying or any other action taken in respect of this e-mail is prohibited and may be unlawful. If you are not the intended recipient, please notify the sender immediately by using the reply function and then permanently delete what you have received

From: Tom Marshall [mailto:t.p.marshall@bham.ac.uk]
Sent: 28 May 2009 09:31
To: NRES Queries Line
Subject: One page summary of evaluation

Dear Hugh

As we discussed last week I have attached a one page summary of the evaluation of the evaluation of targeted case finding in primary care. I trust this will be sufficiently clear so that you can write a letter indicating that the project does not need ethical approval, but if not please get back in touch.

Thank you.

Yours sincerely

Tom Marshall

Dr. Tom Marshall, MRCGP, FFPH, PhD,
Director of MPH Programme,
Unit of Public Health, Epidemiology and Biostatistics,
University of Birmingham,
Edgbaston,
Birmingham,
B15 2TT,
U.K.

Tel: +44 (0)121 414 7832
Fax: +44 (0)121 414 7878

P **Please consider the environment before printing this e-mail**

From: Tom Marshall
Sent: 22 May 2009 22:17
To: NRES Queries Line
Subject: RE: Query about the need for NRES approval

Dear Hugh

Thank you for the very helpful telephone conversation on Thursday. As we discussed I will send you a 1 page summary of the evaluation so that you can write a letter indicating that the project does not need ethical approval.

Thank you

Tom Marshall

From: NRES Queries Line [mailto:queries@nres.npsa.nhs.uk]
Sent: Wed 06-May-09 10:06 AM
To: Tom Marshall
Subject: RE: Query about the need for NRES approval

Your query was reviewed by our Queries Line Advisers.

Yes you can call him on the number provided.

I hope this helps.

Regards

Streamline your research application process with IRAS (Integrated Research Application System). To view IRAS and for further information visit www.myresearchproject.org.uk

---

Queries Line
National Research Ethics Service
National Patient Safety Agency
4-8 Maple Street
London

W1T 5HD

Website: www.nres.npsa.nhs.uk
Email: queries@nres.npsa.nhs.uk

Ref:
**

Help save paper - do you need to print this email?
This e-mail (and any files transmitted with it) is intended for the recipient only. It may contain confidential information and may be protected by law as a legally privileged document and copyright work; its content should be not disclosed, forwarded or copied. If you are not the intended recipient, any reading, printing, storage, disclosure, copying or any other action taken in respect of this e-mail is prohibited and may be unlawful. If you are not the intended recipient, please notify the sender immediately by using the reply function and then permanently delete what you have received

From: Tom Marshall [mailto:t.p.marshall@bham.ac.uk]
Sent: 06 May 2009 10:04
To: NRES Queries Line
Subject: RE: Query about the need for NRES approval

Thank you – is it OK to call him?

Tom Marshall

Dr. Tom Marshall, MRCGP, FFPH, PhD,
Director of MPH Programme,
Unit of Public Health, Epidemiology and Biostatistics,
University of Birmingham,
Edgbaston,
Birmingham,
B15 2TT,
U.K.

Tel: +44 (0)121 414 7832
Fax: +44 (0)121 414 7878

P **Please consider the environment before printing this e-mail**

From: NRES Queries Line [mailto:queries@nres.npsa.nhs.uk]
Sent: 06 May 2009 09:58
To: Tom Marshall
Subject: RE: Query about the need for NRES approval

Your query was reviewed by our Queries Line Advisers.

Contact details for Dr Hugh Davies, Ethics Adviser are below.

Email: hugh.davies@nres.npsa.nhs.uk

Mobile: 07979 771 284

Regards

Streamline your research application process with IRAS (Integrated Research

Application System). To view IRAS and for further information visit www.myresearchproject.org.uk

Queries Line
National Research Ethics Service
National Patient Safety Agency
4-8 Maple Street
London
W1T 5HD

Website: www.nres.npsa.nhs.uk
Email: queries@nres.npsa.nhs.uk

Ref: 04/02
**

Help save paper - do you need to print this email?
This e-mail (and any files transmitted with it) is intended for the recipient only. It may contain confidential information and may be protected by law as a legally privileged document and copyright work; its content should be not disclosed, forwarded or copied. If you are not the intended recipient, any reading, printing, storage, disclosure, copying or any other action taken in respect of this e-mail is prohibited and may be unlawful. If you are not the intended recipient, please notify the sender immediately by using the reply function and then permanently delete what you have received

From: Tom Marshall [mailto:t.p.marshall@bham.ac.uk]
Sent: 05 May 2009 13:42
To: NRES Queries Line
Subject: RE: Query about the need for NRES approval

To whom it may concern,

I need some clarification.

You have advised that I can speak to someone next week. But I am not clear who I should telephone and there is no name or telephone number at the bottom of the email address. Can you tell me who I should speak to and what number I should telephone?

I was aware that the qualitative research will require ethical approval, but I am unclear what aspect of the implementation of this prevention programme makes it necessary to seek ethical approval.

In one PCT, funding was allocated through NIHR to evaluate this programme at a time when the PCT was already recruiting staff to implement the programme. This is typical of programmes being evaluated under the NIHR CLAHRC scheme, where implementation is resourced by the NHS and evaluation is funded by NIHR. Implementation and evaluation are therefore working to different timetables. The practical effect of this is that since late April 2009 the programme is already being implemented in some practices in one PCT. Those practices in which it is being implemented are designated as the intervention group, those where implementation will take place later in the year will be the control group. I need to discuss the implications of this for ethical approval.

Yours sincerely

Tom Marshall

Dr. Tom Marshall, MRCGP, FFPH, PhD,
Director of MPH Programme,
Unit of Public Health, Epidemiology and Biostatistics,
University of Birmingham,

Edgbaston,
Birmingham,
B15 2TT,
U.K.

Tel: +44 (0)121 414 7832
Fax: +44 (0)121 414 7878

P Please consider the environment before printing this e-mail

From: NRES Queries Line [mailto:queries@nres.npsa.nhs.uk]
Sent: 05 May 2009 13:03
To: Tom Marshall
Subject: RE: Query about the need for NRES approval

Your query was reviewed by our Queries Line Advisers.

I'd advise this should be reviewed by an REC as it seems to be a cluster randomised trial. If problems or further advice is required, I'd be happy to discuss next week.

I hope this helps.

Regards

Streamline your research application process with IRAS (Integrated Research Application System). To view IRAS and for further information visit www.myresearchproject.org.uk

Queries Line
National Research Ethics Service
National Patient Safety Agency
4-8 Maple Street
London
W1T 5HD

Website: www.nres.npsa.nhs.uk
Email: queries@nres.npsa.nhs.uk

Ref: 04/02
**

Help save paper - do you need to print this email?
This e-mail (and any files transmitted with it) is intended for the recipient only. It may contain confidential information and may be protected by law as a legally privileged document and copyright work; its content should be not disclosed, forwarded or copied. If you are not the intended recipient, any reading, printing, storage, disclosure, copying or any other action taken in respect of this e-mail is prohibited and may be unlawful. If you are not the intended recipient, please notify the sender immediately by using the reply function and then permanently delete what you have received

-----Original Message-----
From: Tom Marshall [mailto:t.p.marshall@bham.ac.uk]
Sent: 30 April 2009 19:27
To: NRES Queries Line

Subject: RE: Query about the need for NRES approval

To whom it may concern,

I am about to undertake an evaluation of a programme of cardiovascular disease prevention in primary care. I need some advice on which parts of this evaluation require ethical approval.

It is my understanding that the quantitative evaluation should not require ethical approval because patients are treated in accordance with standard practice, no additional data will be collected from patients and the routine data required for evaluation does not include any patient identifiable details. However I believe that the qualitative evaluation will require ethical approval as we intend to interview staff and patients who participate in the programme of cardiovascular prevention.

I would be grateful if you could confirm whether this is the case.

I have attached a brief outline of the study.

Yours sincerely

Tom Marshall

Senior Lecturer in Public Health

University of Birmingham

From: NRES Queries Line [mailto:queries@nres.npsa.nhs.uk]
Sent: Thu 23-Apr-09 10:42 AM
To: Tom Marshall
Subject: RE: Query about the need for NRES approval

Your query was reviewed by our Queries Line Advisers.

So that we might further consider your query, please email an A4 summary (one side only) outlining your proposal to Queries Line. For ease of reference please include your request in the covering email.

I look forward to hearing from you.

Regards

Streamline your research application process with IRAS (Integrated Research Application System). To view IRAS and for further information visit www.myresearchproject.org.uk <http://www.myresearchproject.org.uk/>

National Research Ethics Service
National Patient Safety Agency
4-8 Maple Steet
London
W1T 5HD

Website: www.nres.npsa.nhs.uk <http://www.nres.npsa.nhs.uk/>
Email: queries@nres.npsa.nhs.uk <mailto:jenny.powell@nres.npsa.nhs.uk>

Ref: 02/02

**
This reply may have been sourced in consultation with other members of the NRES team.
***

P Help save paper - do you need to print this email?
This e-mail (and any files transmitted with it) is intended for the recipient only. It may contain confidential information and may be protected by law as a legally privileged document and copyright work; its content should be not disclosed, forwarded or copied. If you are not the intended recipient, any reading, printing, storage, disclosure, copying or any other action taken in respect of this e-mail is prohibited and may be unlawful. If you are not the intended recipient, please notify the sender immediately by using the reply function and then permanently delete what you have received

-----Original Message-----
From: Tom Marshall [mailto:t.p.marshall@bham.ac.uk]
Sent: 22 April 2009 22:12
To: NRES Queries Line
Subject: Query about the need for NRES approval

To whom it may concern,

I am about to undertaking an evaluation of a programme of cardiovascular disease prevention in primary care. I need some advice on which parts of this evaluation require ethical approval.

It is my understanding that the quantitative evaluation should not require ethical approval because there patients are treated in accordance with standard practice, no additional data will be collected from patients and the routine data required for evaluation does not include any patient identifiable details. However I believe that the qualitative evaluation will require ethical approval as we intend to interview staff and patients who participate in the programme of cardiovascular prevention.

I would be grateful if you could confirm whether this is the case.

I have explained this in a bit more detail below and attached a brief outline of the study.

Yours sincerely

Tom Marshall

Senior Lecturer in Public Health

University of Birmingham

CVD PREVENTION PROGRAMME

Essentially this programme involves identifying untreated high risk patients from primary care electronic medical records. A list of these high risk patients is produced within each participating practice. The practice then sends these patients a letter inviting them to attend for cardiovascular assessment in their general practice by either a project nurse (in one PCT) or a project pharmacist (in another PCT). Those who attend are assessed. If necessary they are referred for lifestyle advice (such as smoking cessation or advice on physical activity) and if eligible they are referred to the GP for prescription of drug treatment. A minor variation in one PCT is that the pharmacist is licensed to prescribe and may start treatment.

No patient identifiable information is removed from the practices. In two PCTs software is available which will generate the list of high risk patients. The practices check the list to exclude patients who may have moved away, died or be terminally ill and generate standard invitation letters and information sheets to send to the patients.

All aspects of the assessment, referral for lifestyle advice and treatment follow standard local PCT guidelines. Indeed it has since become Department of Health policy to offer CVD assessment to every adult over 40. The only thing that differs about this service is that we have a specific strategy for inviting patients and a specific member of staff to undertake the assessments.

QUANTITATIVE EVALUATION

We have funding under the NIHR CLAHRC scheme to evaluate this implementation. Three separate programmes are being implemented in two separate PCTs. However the programmes are very similar and we intend to evaluate them in as similar a way as possible in order to be able to pool our results. The evaluation will take the form of a cluster randomised trial comparing the numbers of persons assessed and started on treatment in intervention practices and control practices.

IMPLEMENTATION PLAN

There are not sufficient project nurses (or pharmacists) to implement the programme in all practices at the same time. Practices that have agreed to take part in the programme are randomly allocated by the PCT to receive the

intervention earlier or to receive the intervention later. Those practices where the programme is implemented earlier will be the intervention group. Those were it is implemented later will be the control group.

Intervention Practices

In those practices who receive the intervention earlier the PCT will facilitate the production of a list of their untreated high risk patients and then systematically invite them for assessment.

Control Practices

In those practices who receive the intervention later, the PCT will provide the practice with the equivalent list of their untreated high risk patients but will not provide specific staff to assess these patients until the staff have finished working in other practices. There will therefore be a delay of a number of months before the project nurses (or pharmacists). The exact number of months depends on how quickly high risk patients can be assessed in the intervention practices.

Patients in both intervention and control practices can potentially benefit from the fact that they are provided with a list of untreated high risk patients. In both intervention and control practices if any patients are identified with untreated high blood pressure or other findings that need immediate action, this will be specifically drawn to the attention of the GP.

OUTCOMES AND DATA COLLECTION

To evaluate the implementation we wish to count the number of persons on the target list who undergo for CVD assessment in intervention and control practices; the numbers referred to smoking cessation services, dietetic services or physical activity services; and the numbers started on preventive treatments. The primary outcome is the number started on preventive treatment.

All of the required data is stored in primary care electronic medical records. It is therefore proposed to evaluate the CVD prevention programme by asking for routine data extraction from the participating practices. No patient identifiable information will be included in the data extraction. Only age, sex, prescribing history and cardiovascular risk factors (eg: blood pressure, cholesterol levels, smoking status etc).

QUALITATIVE EVALUATION

We wish to evaluate the experience of the cardiovascular prevention programme from the perspective of participants, staff delivering the programme and other general practice staff. To do this we intend to interview a sample of participants, staff delivering the programme and other general practice staff.

Dr. Tom Marshall, MRCGP, FFPH, PhD,

Director of MPH Programme,

Unit of Public Health, Epidemiology and Biostatistics,

University of Birmingham,

Edgbaston,

Birmingham,

B15 2TT,

U.K.

Tel: +44 (0)121 414 7832

Fax: +44 (0)121 414 7878

P Please consider the environment before printing this e-mail
