# Supplementary material for: Mixed methods evaluation of targeted case finding for cardiovascular disease prevention using a stepped wedged cluster RCT
Source: BMC Public Health. 2012 Oct 26;12:908. doi: 10.1186/1471-2458-12-908 (PMC3505746; doi:10.1186/1471-2458-12-908)
Supplement: Additional file 5 — Consent form. [file 1471-2458-12-908-S5.pdf]

Consent form

Title of study: Investment in prevention (Evaluation of targeted prevention of cardiovascular disease in primary care)

I confirm that I have read and understood the participant information sheet for this study and have had the opportunity to ask questions.

I understand that my participation is voluntary and that I am free to withdraw at any time, without giving any reason, without my legal rights being affected.

I agree to take part in the study.

I confirm that I am happy to have my interview recorded and transcribed.

I confirm that I give permission to use direct quotations.

I understand that I can give or refuse permission to use individual photographs taken as part of the study in reports/publications. I understand that permission to include my photos will be agreed on a photo-by-photo basis after the interview.

Name of Interviewee [please print]__________________________

Signature __________________________ Date _____(day)/_____(month)/____(year)

Name of Researcher [please print]__________________________

Signature __________________________ Date _____(day)/_____(month)/____(year)
