# Supplementary material for: Mixed methods evaluation of targeted case finding for cardiovascular disease prevention using a stepped wedged cluster RCT
Source: BMC Public Health. 2012 Oct 26;12:908. doi: 10.1186/1471-2458-12-908 (PMC3505746; doi:10.1186/1471-2458-12-908)
Supplement: Additional file 7 — Participant information sheet. [file 1471-2458-12-908-S7.pdf]

Participant Information Sheet

Study title: Investment in prevention (Evaluation of targeted prevention of cardiovascular disease in primary care)

You are being invited to take part in a study on a programme being run in general practices on the prevention of cardiovascular disease. This Information Sheet is provided to explain why this evaluation is being done and what it will involve, in order to help you decide if you want to participate. Please take time to read the following information carefully and to discuss it with others if you wish. Ask us if there is anything that is not clear or if you would like more information. Please take time to decide whether or not you wish to take part.

Thank you for reading this.

What is the purpose of the study?

The study has been designed to evaluate how well the programme is working and whether it is appropriate and acceptable for members of the local community.

Why have I been chosen?

We would like you to participate in this study because we wish to seek the views of health professional and patients involved in the programme.

Do I have to take part?

It is entirely up to you to decide whether or not to take part. If you do decide to contribute, you will be given this information sheet to keep and be asked to sign a consent form. You will be given a copy of the consent form to keep. If you decide to take part you are still free to withdraw at any time and without giving a reason.

What will happen to me if I take part?

We wish to interview you about your experiences of attending the risk screening appointment and how you feel more generally about preventive healthcare and how it fits into your daily life.

You will be given a disposable camera to take home and will be asked to take approximately 10-15 photos of aspects of your life that you consider have an impact on your health, happiness and general wellbeing. These can be positive or negative things - anything at all in your life that you think is relevant to your own health and wellbeing. These photos will form the basis of the discussion at the interview.

The interviews will be undertaken by members of the service evaluation team. These are experienced researchers who are trained and qualified in this research method. The interviews will take place at a place agreed by you and the researcher, such as your home or a local community or health centre.

Will my taking part in this study be kept confidential?

The interview data will be kept completely confidential and reported anonymously. Any details that could identify you (such as names or places) will be removed before the study is reported or published. The interviews will be recorded and transcribed (a written copy will be made). In line with the University of Birmingham's Code of Conduct for Research, the interview transcripts will be preserved and accessible for ten years after publication of the study's findings. The transcripts will not identify the interviewees by name.

What will happen to the results of the study?

The results will be used to inform future developments of programmes for prevention of disease in the local NHS. Key findings from the study will be published in relevant scientific or professional journals.

Who is organising and funding the service evaluation?

The service evaluation is being organised by the University of Birmingham and funded by the local Primary Care Trust and the National Institute for Health Research.

What indemnity arrangements are in place?

This study is covered by the University of Birmingham's insurance policy for negligent harm. The study is not covered for non-negligent harm, as this is not included in the University of Birmingham's standard insurance policy.

How can I get further information?

Please ask Nicola Gale (n.gale@bham.ac.uk, 0121 414 9089) if you would like to get any more information about the study or to let her know that you would like to take part.

Department of Primary Care Clinical Sciences
School of Health and Population Sciences
University of Birmingham
Birmingham
B15 2TT

Thank you for your help.
