# Supplementary material for: Mixed methods evaluation of targeted case finding for cardiovascular disease prevention using a stepped wedged cluster RCT
Source: BMC Public Health. 2012 Oct 26;12:908. doi: 10.1186/1471-2458-12-908 (PMC3505746; doi:10.1186/1471-2458-12-908)
Supplement: Additional file 4 — Application for ethical review ERN_10-0429. [file 1471-2458-12-908-S4.pdf]

UNIVERSITY OF BIRMINGHAM

Research and Commercial Services

Dr J H Wilkie
BSc(Hons) PhD MBA CEng MIMMM

7th June 2010

Dr Nicola Gale and Dr Tom Marshall
School of Health and Population Sciences
University of Birmingham

Dear Dr Gale and Dr Marshall

Re: "Investment in prevention (evaluation of targeted prevention of cardiovascular disease in primary care)"
Application for Ethical Review ERN_10-0429

Thank you for your application for ethical review for the above project, which has now been reviewed by the Life and Health Sciences Ethical Review Committee.

It is understood that this project would not require ethical review via the National Research Ethics Service (NRES) as it has been classified as service evaluation rather than research.

On behalf of the Committee, I am pleased to confirm a favourable ethical opinion for your project, subject to the Committee's satisfaction with your response to the following conditions:

- In your application, you state that if a participant withdraws, their data will be automatically included in the study. If this is the case, it should be very clearly stated within the participant information sheets. For the patient participants, it should also be clearly explained what will happen to any photographs, if a participant withdraws after the photographs have been submitted.

- In the consent form, you request permission to use direct quotations. It should be clarified in the consent form that these quotations will be anonymised, and the proposed use of direct quotations should be explained in the participant information sheets.

- Please contact the relevant NHS R&D department in order to confirm they are happy with the arrangements in place to interview NHS staff.

Institute of Research Development Birmingham Research Park
University of Birmingham Edgbaston Birmingham B15 2SQ United Kingdom
T: +44 (0)121 414 5712 F: +44 (0)121 414 7151 W: www.bham.ac.uk

INVESTOR IN PEOPLE

- A Sponsor should be identified for this study, in line with the requirements of Department of Health's Research Governance Framework. Please clarify who will be sponsoring this study, and if necessary please complete and return the attached PF1 form to request that the University acts as Sponsor.

I would be grateful if you could confirm by email to s.l.cottam@bham.ac.uk that these conditions will be met, and also provide the requested information and documentation prior to the commencement of the study.

I would like to remind you that any substantive changes to the nature of the study as described in the Application for Ethical Review, and/or any adverse events occurring during the study should be promptly bought to the Committee's attention by the Principal Investigator and may necessitate further ethical review.

Please confirm receipt by return email.

Yours sincerely

pp. **Dr Jane Steele**
Chair
Life and Health Sciences Ethical Review Committee

UNIVERSITY OF BIRMINGHAM

Application for the University of Birmingham to Act as Sponsor under the Department of Health's Research Governance Framework for a <u>research study</u> – <u>PF1</u>

The University is requested to agree to act as Sponsor for <u>staff led research</u> or <u>supervised-postgraduate student research study</u> falling under the Research Governance Framework for Health and Social Care with respect to the following project:

PLEASE CONSULT THE NOTES FOR APPLICANTS BEFORE USING THIS FORM

**Project Title:**	
**Chief/Principal Investigator (Academic Supervisor in case of supervised student research):**	
**Employment Status (whether UoB employee or on honorary position)**	
**School/Division:**	
**Email Address and Phone:**	
**Is this a postgraduate research student project?**	**Y/N**
**Name of Student and degree course (where appropriate):**	
**Who is Funding the research?**	
**What is stage of funding?**	**Application/awarded**
**Finance Office Account Number:**	
**Site of Research:**	
**Provide details whether the research requires access to NHS patients, their data or tissues or NHS staff**	
**Identify briefly the nature of study:**	
**Is the Research <u>solely</u> based upon one or a combination of the following: questionnaires or access to human cells or tissue or measurements of physiological processes or collections of body secretions by non-invasive methods or the administration mouth of foods or nutrients or variation of diet (other than the administration of drugs or other foods supplements) or venopuncture?**	**Y/N**
**If this is a collaborative project, is the University leading it?**	
**Identify the scientific peer-review body:**	
**Identify the Research Ethics Committee that has reviewed the proposal and Reference Number: (Attach copy of ethics opinion if available)**	

Which are the external bodies requiring a letter regarding the Sponsor role?	

DECLARATIONS

Declaration by Principal/Chief Investigator/Academic Supervisor (*)

I, **Name**.., as Principal/Chief Investigator/Academic Supervisor (*delete as appropriate) for **Project Title:**
....
.... confirm that:

1. The information in this form is accurate to the best of my knowledge and belief and I take full responsibility for this research project.
2. I understand the duties required of the Investigators, the Funders and the Sponsor by the Research Governance Framework and undertake the duties of Principal or Chief Investigator.
3. I undertake to comply with the University's policies and procedures and the principles of the Department of Health's Research Governance Framework and the Medicines for Human Use (Clinical Trials) Regulations 2004.
4. To the best of my knowledge the School is properly equipped to take on the role of Sponsor.
5. I take responsibility for ensuring that all staff and students involved in this research are familiar and abide with the Research Governance Framework and the University's relevant policies and guidelines.
6. I understand and agree that the study files, records data and documents may be subjected to inspection for auditing and monitoring purposes.
7. I understand that information relating to this research, and about me as a researcher, will be held by the Research Governance Officer and on the Research Governance Database. This information will be managed according to the principles established in the Data Protection Act 1988.

Signed ....

Name .... Date ....

Declaration by Head of School/Division

I, **Name:**..., in my capacity as
Title: ....confirm that:

I have read and understood the roles of the Investigators, Funders and Sponsors. The School/Division is properly equipped to carry out the role(s) of Sponsor including monitoring the project as appropriate. I hereby recommend that the University accepts the role of Sponsor for the above project.

Signed ....

Name .... Date ....

For Postgraduate Research Student Projects:

Declaration by the Research Student and Principal Investigator

I, **Name:**……………………………………………………………………., as the research postgraduate student conducting the research for the above project confirm that:

1. The information in this form is accurate and to the best of my knowledge and belief and I take full responsibility for my role in this research project.
2. I understand the duties required of the Investigators, the Funders and the Sponsor by the Research Governance Framework and undertake the duties of Principal or Chief Investigator.
3. I undertake to comply with the University's policies and procedures and the principles of the Department of Health's Research Governance Framework.
4. I understand and agree that the study files, records data and documents may be subjected to inspection for auditing and monitoring purposes.
5. I understand that information relating to this research, and about me as a researcher, will be held by the Research Governance Officer and on the Research Governance Database. This information will be managed according to the principles established in the Data Protection Act 1988.

Signed ……………………………..

Name …………………………….. Date ……………………………..

E-mail address ……………………………………………………………………...
